# The effect of family supportive supervisor behavior on teachers’ innovative behavior and thriving at work: A moderated mediation model

**DOI:** 10.3389/fpsyg.2023.1129486

**Published:** 2023-03-08

**Authors:** Qiu Li, Minghui Liu

**Affiliations:** School of Public Finance and Taxation, Dongbei University of Finance and Economics, Dalian, China

**Keywords:** family-supportive supervisor behavior, work-family enrichment, teacher innovative behavior, thriving at work, proactive personality

## Abstract

**Objective:**

In today’s society, teachers are increasingly focused on the harmonious development of work and family. However, few studies have discussed family supportive supervisor behavior to promote teachers’ innovative behaviors and thriving at work. The study explores the mechanisms of family-supportive supervisor behaviors on teachers’ innovative behaviors and thriving at work.

**Methods:**

In this study, we adopt a questionnaire-based follow-up study of 409 career married teachers in Northwest China at three time points based on the Work-home Resource Model and Resource Conservation Theory.

**Results:**

The results indicate that family supportive supervisor behavior has a significant positive predictive effect on teachers’ innovative behavior and thriving at work, and work-family enrichment mediates between this relationship. In addition, proactive personality moderates the relationship between family-supportive supervisor behavior and work-family enrichment and the mediating role of work-family enrichment.

**Conclusion:**

Prior research has focused more on the impact of job characteristics within the work domain on work innovation behavior and thriving at work, and some studies have explored the impact of family-level factors on teacher behavior, but more often than not, they have been described based on a conflict perspective. This paper explores the positive impact of family-supportive supervisor behavior on teachers’ innovative behaviors and thriving at work from a resource flow perspective and identifies its potential boundary conditions. This study extends theoretical research on family-work relationships while providing new grounding and research perspectives for improving teacher work and family enrichment.

## Introduction

In recent years, the conflict between teachers’ work and family roles has intensified with the rapid economic development and the continuous advancement of Internet technology. To effectively mitigate the adverse effects of this conflict, companies have proposed family support policies such as flexible work schedules and paid leave. However, these policies have not worked as well as expected. The reason for the less-than-ideal results lies in the lack of deeper and more effective knowledge and communication of the policies’ implementation by supervisors. As a result, family supportive supervisor behavior (FSSB), which supports teachers in their family responsibilities, has received widespread attention from administrators and scholars (Crain and Stevens, 2018). The purpose of this supervisor behavior is to balance the teacher’s work-family relationship and to achieve a “win-win” situation for both teachers and the organization. Research has shown that FSSB not only stimulates teachers’ organizational commitment, performance, and reduces turnover, but also enhances teachers’ marital satisfaction, subjective wellbeing, and job satisfaction, as well as their physical and mental health (Olde-Dusseau, 2012; Aryee et al., 2013; Olde-Dusseau et al., 2016; Hammer et al., 2019; Lu et al., 2019; Talukder, 2019; Chen and Zhang, 2020; Liu et al., 2020; Rofcanin et al., 2020; Shi et al., 2020; Luo et al., 2022; Susanto et al., 2022; Ahmed et al., 2023; Kim et al., 2023). In the current context and traditional Chinese culture, does family-supportive supervisor behavior influence the level of thriving at work and teachers’ innovative behaviors, allowing employees to “go to work with passion and innovative thinking” and thereby promoting organizational productivity? What factors play a key role in the process? All these problems need to be explored in depth.

Reviewing previous studies, scholars have focused more on the effects of thriving at work and teacher innovation behavior within the work domain, suggesting that thriving at work and teacher innovation behavior is effective in alleviating burnout that occurs at work (Hildenbrand et al., 2018) and enhancing creative performance (Christensen-Salem et al., 2021). In addition, thriving at work and teacher innovative behavior on job satisfaction (Chang and Busser, 2020; Qaiser et al., 2020; Jiang et al., 2021), life satisfaction (Zhai et al., 2020) organization commitment (Porath et al., 2012; Abid et al., 2019) have a positive effect and a negative effect on turnover intentions (Chang and Busser, 2020). In recent years, some scholars have begun to study the positive effects of family domain on thriving at work and teacher innovation behavior, but they are limited to the effects of family domain and family-related policies on thriving at work and teacher innovation behavior, and fewer scholars have focused on the positive effects of family-supportive supervisor behavior on thriving at work and teacher innovation behavior (Carmeli and Russo, 2016).

According to the work-home resource model, individuals are able to achieve simultaneous participation in multiple role activities. When individuals are engaged in work or family roles, they instinctively use the resources generated by the roles to stimulate their potential and then promote their own development, and apply the experience gained while working on other activities (Ten Brummelhuis and Bakker, 2012; Du et al., 2020). That is, there is a positive mutual spillover relationship between work and family (Kopperud et al., 2020; Aw et al., 2021; Deng et al., 2021). Researchers have argued that work-family enrichment, which refers to the beneficial impact of the experiences and resources gained from employees’ involvement in work matters on the quality of family life, can best represent the mechanisms inherent in a positive work-family interface (Greenhaus and Powell, 2006; Gopalan et al., 2021; Shen et al., 2022). Resources representing one domain help develop personal resources that drive increased outcomes in another domain (Ten Brummelhuis and Bakker, 2012). Enrichment are formed when individuals accumulate family-supportive work resources that allow individuals to perform better at work. Resource formation is a key driver of the enrichment process because it is an asset that individuals need to draw upon when faced with problems (Wayne et al., 2007). Therefore, if family-supportive supervisor behavior have a positive impact on work flourishing and innovative teacher behavior, work-family enrichment should be a necessary and critical component of this positive effect.

In addition, in the study of organizational behavior, the influence of individual traits of employees deserves attention. At present, researchers mostly focus on the positive role of organizational environmental characteristics between family and work, and seldom discuss the impact of individual proactive personality differences on resource spillover (Hammer et al., 2013; Matthews et al., 2014). Proactive personality is a positive and stable personality trait. Individuals with this trait can always seize the opportunity to change their environment and are willing to take action (Bateman and Crant, 1993; Li et al., 2020; Yi-Feng Chen et al., 2021). Studies have shown that employees with higher levels of proactive personality are less bound to the outside world and feel more motivated by positive events in their lives, and tend to exchange information and share knowledge with others (Horng et al., 2016; Abid et al., 2021). Therefore, proactive personality may play a moderating role in the indirect relationship between family-supportive supervisor behavior and thriving at work and teachers’ innovative behavior through work-family enrichment.

## Theory and hypothesis

### Family supportive supervisor behavior and teachers’ innovative behavior

Family supportive supervisor behavior refers to the behaviors exhibited by supervisors to help teachers fulfill their family responsibilities, including emotional concern for teachers’ family life, provision of instrumental resources for teachers, readjustment of working hours, and exemplary behaviors exhibited by teachers to balance work-family relationships (Hammer et al., 2013; Yu et al., 2022). According to resource conservation theory, when facing the threat of loss of their own resources, individuals tend to adopt a conservative way to preserve resources and refuse risky activities. Conversely, individuals are psychologically more motivated when resources are available, and they proactively acquire resources and combine them to create greater value. Teachers’ innovative behavior is resource-depleting (Zhang and Bartol, 2010; McKersie et al., 2019). The process of innovation from idea generation to implementation requires teachers to invest considerable time, cognitive and emotional resources, and to be prepared for the risks of failure and rejection by their peers (Zhang and Bartol, 2010; Zhou et al., 2022). If schools want teachers to actively participate in innovation, they need to enrich resources for teachers and make them actively participate in innovation.

In summary, based on resource conservation theory, we believe that FSSB promotes teachers’ innovative behavior in several ways. First, FSSB can help teachers fulfill their family responsibilities, reduce the drain on teachers’ resources from negative events such as work-family conflict and family stress. It enables teachers to devote themselves to thinking and solving problems creatively in their work (Swanberg et al., 2011; Yu et al., 2022). Secondly, the concern and support shown by supervisors for teachers’ family life helps to create a safe working atmosphere, which enables teachers to dare to try new methods and procedures, and then actively innovate (Carmeli and Russo, 2016; Zhining et al., 2020). Thirdly, FSSB helps teachers to live their family life in a flexible way, so that they can meet their family (work) responsibilities without giving up their work (family). This allows teachers to interact not only with supervisors and colleagues, but also with family members in depth, further broadening their horizons resources and improving their innovation (Aleksic et al., 2017; Erdogan et al., 2022). Finally, based on the perspective of social exchange theory, teachers give back behaviors because the organization provides them with valuable resources that meet their needs for resources in their work and life. These behaviors further enhance trust and commitment with the supervisor, who will be willing to provide and supplement the resources needed for the teacher’s innovation. According to the principle of reciprocity, teachers will actively engage in innovative behaviors to accomplish their goals in work and life. Accordingly, this paper proposes the following hypothesis.

Hypothesis 1: Family supportive supervisor behavior has a significant positive affect on teachers’ innovative behavior.

### Family supportive supervisor behavior and teacher’s thriving at work

Thriving at work refers to the positive psychological state of learning and vitality that accompanies the work process (Spreitzer et al., 2005). It not only provides a sense of enjoyment but also leads to productivity. Research shows that teachers and departments that thrive at work perform better and earn more (Roshan and Arulrajah, 2021). At the same time, increasing personal resources (e.g., self-control, positive affective traits, and self-esteem) and work resources (e.g., mini-breaks, leadership support, etc.) can significantly increase teachers’ thriving at work. Family-supportive supervisor behavior is a leadership-supportive behavior and an important resource for family support. Therefore, we combine the connotative and structural dimensions of family-supportive supervisor behavior in this paper to illustrate how it motivates teachers to flourish at work along three pathways.

First of all, emotional support and instrumental support in family-supportive supervisor behavior provide teachers with sufficient and effective resources. In order to realize the value-added of existing resources, individuals will take action to accumulate resources and show their due vitality and learning state. As far as vitality is concerned, this behavior can inhibit the loss of teachers’ work energy caused by work-family conflict, so that they have sufficient work energy (Jiang et al., 2015; Shi et al., 2019). At the same time, supervisors’ understanding of teachers’ family needs helps to establish a close and harmonious interactive relationship between teachers and supervisors, which is an important source of teachers’ vitality (Spreitzer et al., 2005; Talukder and Galang, 2021). In addition to enhancing teachers’ vitality, FSSB can also promote teachers’ learning. It is an important part of FSSB to provide teachers with strategies and experiences to coordinate the relationship between work and family. By learning and applying these knowledge and skills, teachers can effectively respond to the demands of work and family, balance them, and thereby feel enhanced and developed.

Secondly, family-supportive supervisor behavior not only provide resources, but also create a pro-family organizational culture and atmosphere. When the basic resource needs of individuals are met, the perceived organizational climate will increase their psychological capital, enhance the mutual trust and commitment between supervisors and teachers, and promote the continuous improvement of exchange relations. Teachers are motivated to “bring their passion to work” in return for their supervisors’ efforts.

Thirdly, the innovative work-family management dimension of family-supportive supervisor behavior includes the change of working time, place and mode, which not only embodies flexibility, but also embeds the concept of “win-win.” It pays attention to innovation and emphasizes strategy, which can not only balance the work and family of teachers, but also make organizations and individuals mutually beneficial. It has changed the previous situation of focusing only on individual needs and neglecting organizational interests, or tending to solve family conflicts and neglecting work status and performance. Therefore, based on the above contents, this study proposes the following hypotheses:

Hypothesis 2: FSSB has a significant positive affect on teacher’s thriving at work.

### The mediating role of work-family enrichment

Work-family enrichment effectively illustrate the intrinsic positive mechanisms of the work-family interface (Heskiau and McCarthy, 2021). According to Greenhaus and Powell (2006), work-family enrichment is the degree to which resources in one role field permeate across borders and promote performance and improve quality of life in another role field. Work-family enrichment includes two dimensions: Work-family enrichment and family-work enrichment (Chan et al., 2020). Work-family enrichment refers to the extent to which work experience and resources can improve the quality of family life, while family-work enrichment refers to the extent to which family experience and resources can improve the quality of work and life (Koekemoer et al., 2020). This article focuses on the effects of family-supportive supervisor behavior provided by organizations on the quality of life of individuals’ families, selecting work-family enrichment as the main variable for the study.

In the organization, the supervisor’s general support for teachers is mainly reflected in the support and resources given to individual work, but not necessarily including the support for individual family. Moreover, the family-friendly resources derived from work often come from work structures and work processes, such as flexible work time rotation and flexible policy support. In terms of the meaning of family support supervisor behavior, it includes not only the support given by work, but also the satisfaction of family needs, including the flexible arrangement of working time and schedule, as well as the adjustment according to individual differences in the implementation of family support. It allows teachers to better meet the needs of their families while being able to successfully complete their work assignments. Based on resource conservation theory, the resources provided by the work domain are the basis for work-family enrichment. Therefore, family supportive supervisor behavior, as an important support resource, positively affects teachers’ family and work. Hammer et al. (2013) found that family supportive supervisor behavior has a significant negative effect on employees’ tendency to leave and work-family conflict, and a significant positive effect on job satisfaction and positive work-family spillover. Meanwhile, family supportive supervisor behavior is more significantly related to the above outcome variables than general supervisor support.

In addition, according to the theory of resource preservation, supervisors provide teachers with support resources to meet their individual needs, which can be applied to the participation of family roles, improve the quality of family and life, and realize the cross-border gains of work to family (Carlson et al., 2019). Individuals who want to experience the continuous gain of work to family need to invest time and energy in the role of work and accumulate resources continuously, which is in line with the resource acquisition spiral in the theory of resource preservation, that is, the initial resource acquisition will lead to more resources later (Kalliath et al., 2020). Timms et al. (2015) shows that when teachers feel the positive impact of work and family, they will be more enthusiastic about their work. Cinamon and Rich (2002) show that those teachers who feel the positive impact of work and family show greater vitality and creativity in their work. The mediating effect of work-family enrichment on the relationship between work support resources and thriving at work and innovation behavior has also been confirmed by relevant studies. Therefore, work-family enrichment is an important link between family-supportive supervisor behavior and teacher innovation behavior and thriving at work, and plays a mediating role. Therefore, we make the following assumptions:

Hypothesis 3A: Work-family enrichment mediates the relationship between family-supportive supervisor behavior and teacher innovation behavior.

Hypothesis 3B: Work-family enrichment mediates the relationship between family-supportive supervisor behavior and teacher’s thriving at work.

### The moderating effect of proactive personality

Proactive personality is a stable personality trait with obvious individual differences, which refers to a behavioral tendency to focus on creating or changing the environment (Bateman and Crant, 1993). There is a self-regulatory mechanism between individual trait factors and behavioral outcomes, including the role of proactive personality, which affects the individual’s choice of work-family conflict management. For example, studies have shown that proactive personality has a significant moderating effect between work-family conflict and turnover intention (Bergeron et al., 2014). According to psychological stress theory, if people have different cognitive evaluations of the same event, their attitudes and behaviors will differ and the outcomes will be significantly different (Zhao et al., 2019). Proactive personality, as a positive psychological trait, can affect people’s cognitive evaluation and attitude and behavior when facing stressful events. If individuals have positive psychological traits, they can not only correctly recognize work and family problems, but also flexibly deal with them, and are not easily affected by changes in the internal and external environment (Zhao et al., 2019).

On the basis of resource conservation theory, because individuals have limited resources, they need to make decisions about when, where, and how to allocate resources accordingly. It was found that individual traits influence individual resource allocation. Individuals with high levels of proactive personality tend to be flexible in switching resources between work and family. This shift helps to facilitate work-family relationships (Boyar and Mosley, 2007). According to the work-home resource model, individuals make full use of the resources provided by family-supportive supervisory behaviors when facing work matters. However, both the resources generated by family-supportive supervisor behavior and the employee’s proactive personality are effective scarce resources at the disposal of the individual. To some extent, the two resources can complement or replace each other. Even if the organizational contextual resources provided by family-supportive supervisor behavior are not sufficiently supportive, employees with high levels of proactive personality can effectively enhance work-family gains through optimal allocation of resources, which in turn promotes thriving at work and innovative teacher behavior. Individuals with low levels of proactive personality, on the contrary, will undoubtedly suffer from a lack of resources to facilitate effective work-family transitions, coupled with a lack of psychological resources for thriving at work and innovative work behavior.

Studies have shown that when individuals have a high level of proactive personality, they are not easily bound by the outside world and can always seize opportunities to actively change the environment. If the organization provides resources, individuals will work hard to complete their tasks. Faced with the same situation, individuals with low initiative personality tend to passively accept the environment and are unwilling to make more efforts to deal with work affairs (Bergeron et al., 2014). Proactive personality is also a coping resource for individuals to alleviate role ambiguity and role overload. Individuals with high proactivity are better able to construct and access work resources, have a more optimistic assessment of their ability to change the environment they are in, address role stress in a more effective way, meet role expectations, create a work environment that is beneficial to them, and advance inter-work and family gains (Van den Heuvel et al., 2015). Therefore, the coupling of family-supportive supervisor behavior and proactive personality can effectively enhance the work-family enrichment of employees. To sum up, this study proposes the following hypotheses:

Hypothesis 4: Proactive personality moderates the relationship between family-supportive supervisor behavior and work-family enrichment. That is, the higher the proactive personality, the stronger the positive predictive effect of family-supportive supervisor behavior on work-family enrichment, and the weaker the opposite.

Proactive personality not only moderates the relationship between family-supportive supervisor behavior and work-family enrichment, but also may influence the indirect effect of family-supportive supervisor behavior on teachers’ innovative work behavior and teachers’ thriving at work through work-family enrichment. Specifically, work-family enrichment mediates the effect of family-supportive supervisor behavior on teachers’ innovative work behavior and teachers’ thriving at work, and the level of proactive personality affects the mediating effect. Based on the above reasoning, this study proposes the following hypotheses:

Hypothesis 5A: Proactive personality moderates the indirect effect of family-supportive supervisor behavior on teachers’ innovative work behavior, that is, the higher the proactive personality, the stronger the indirect effect of family-supportive supervisor behavior on teachers’ innovative behavior through work-family enrichment, and vice versa.

Hypothesis 5B: Proactive personality moderates the indirect effect of family-supportive supervisor behavior on teachers’ thriving at work, that is, the higher the proactive personality, the stronger the indirect effect of family-supportive supervisor behavior on teachers’ thriving at work through work-family enrichment, and vice versa.

**FIGURE 1:**
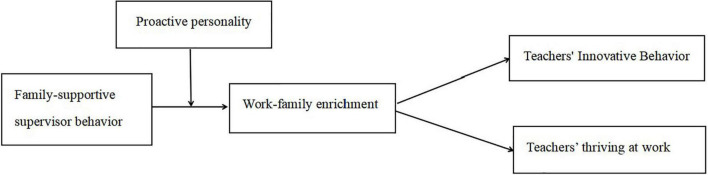
Theoretical model.

In summary, the theoretical model of this study is shown in Figure 1.

## Materials and methods

### Research subjects and data collection

This study conducts a questionnaire survey of teachers in three general higher education schools in southern China. The survey respondents are front-line teaching staff within the schools. To reduce the effect of common method bias on the relationship between variables, the following control methods are used: (1) data are collected in three stages, with a 2 week interval between each two stages; (2) anonymous responses are used to reduce respondents’ tendency to personal bias; (3) variable names are not marked in the questionnaire to conceal the purpose of the study and the meaning of the question items, ensuring that respondents answer the questions according to their true personal feelings. Specifically, the research team placed the questionnaires in small, anonymous, sealable envelopes, each with double-sided tape attached in advance. Before the survey began, supervisors from various departments at each university emphasized the benefits of the study to the faculty members, gave a detailed description of the process, expressed their support for the project, and encouraged active collaboration before the supervisors left the room. The research team then handed out questionnaire envelopes to the subjects. The teachers completed and returned the envelope and returned the questionnaire directly to the researcher; finally, they received a payment of $10 from the researcher. It is also important to note that the questionnaires were paired using a coded format (i.e., the first phase of the questionnaire had a number set on it and subjects were asked to remember their number, and in the next two phases, subjects were asked to fill in their questionnaire number). Throughout the data collection process, the questionnaire was completely anonymous and participation was voluntary.

The survey consists of three phases: in the first stage, teachers evaluated family supportive leadership behavior and teacher proactive personality, and filled in demographic information (gender, age, education level, and working years); in the second stage, teachers evaluated job requirements and work-family gains; in the third stage, teachers evaluated intrinsic motivation, teachers’ innovative work behavior and teachers’ thriving at work. A total of 479 questionnaires were distributed in all three stages, with 476 questionnaires collected in the first stage, 469 questionnaires in the second stage and 453 questionnaires in the third stage. The questionnaires in the three stages were matched, and finally 409 valid questionnaires were obtained. According to the descriptive statistical results of the sample, 52.5% were male, 47.5% were female; 57.46% were under 35 years old, 23.96% were between 36 and 45 years old, 18.34% were over 45 years old; 44.01% were junior college and below, 55.99% were undergraduate and above; 71.88% of them have worked for 5 years or less, 18.83% for 5–10 years and 9.05% for 10 years or more. The questionnaires in the three stages were matched, and 409 valid questionnaires were finally obtained. According to the descriptive statistics of the sample, 52.5% were male and 47.5% were female; 57.46% were under 35 years old, 23.96% were 36 ∼45 years old, and 18.34% were over 45 years old; 44.01% were college and below, 55.99% were bachelor and above; 71.88% had worked for 5 years and below, 18.83% for 5∼10 years, and 9.05% for 10 years and above.

### Variables

In this study, the job demand scale was scored by Likert 5-point method, with 1 representing “very small” and 5 representing “very large.” All other scales were scored using a Likert 7-point scale, with 1 representing “strongly disagree” and 7 representing “strongly agree.” The scales used in this study were all developed by domestic and foreign scholars (see Appendix). In order to make the translated Chinese questionnaires consistent with or similar to the original English questionnaires, the translation and back-translation were conducted by members of the research team and English majors.

#### Thriving at work

The Thriving at Work Scale consists of two dimensions: Vitality and learning. The vitality dimension is based on the scale developed by Atwater and Carmeli (2009) and contains eight items, such as “I have plenty of energy to do my job;” the learning dimension is based on the scale developed by Carmeli and Spreitzer (2011) and contains three items, such as “The new things I learn at work help me in my life.” As the current mainstream measurement instrument, the reliability of the scale has been validated in many research measurements with samples involving members of different types of organizations, which shows that the scale has a relatively wide applicability. In this study, the reliability coefficient of this scale was 0.916.

#### Proactive personality

The scale developed by Seibert et al. (1999) was used and contained 10 items, such as “I am constantly looking for new ways to improve my current life.” The reliability of the scale has been validated in previous studies and has wide applicability. In this study, the reliability coefficient of the scale was 0.901.

#### Innovative behaviors

The innovative behaviors of teachers were measured by using the teacher innovation behavior questionnaire developed by Zhang and Zhang (2012). The questionnaire included three dimensions, including willingness to innovate (4 items), innovative actions (6 items), and innovative outcomes (6 items), with a total of 16 items. The reliability of the scale has been validated in previous studies and has wide applicability. In this study, the reliability coefficient of the scale was 0.942.

#### Work-family enrichment

The scale developed by Wayne et al. (2004) was used, with four items, such as “Having a good job allows me to play the role of a partner better at home.” The reliability of the scale has been validated in previous studies and has wide applicability. In this study, the internal consistency coefficient of this scale was 0.842.

#### Family supportive supervisor behaviors

The scale developed by Hammer et al. (2009) was used, with four items. Hammer et al. (2009) concluded that family supportive supervisor behaviors are mainly related to the leader’s supportive behaviors toward the employees’ family life, and that the strength of their effectiveness is mainly derived from the employees’ perceptions, so they should fill in the items themselves. An example question is “My supervisor demonstrates effective behaviors regarding how to balance work and non-work issues.” The reliability of the scale has been validated in previous studies and has wide applicability. In this study, the internal consistency coefficient of this scale was 0.844.

### Control variable

This study controls for demographic variables such as gender, age, education level, and years of experience in the workforce that may affect the study results. Moreover, previous studies have found that job requirements affect work-family relationships (Bakker et al., 2008), and internal motivation affects thriving at work (Menges et al., 2017). For this reason, the present study also controlled these two variables. Job requirements were measured using a scale developed by Karasek (1979), with questions such as “To what extent are your job requirements in conflict?” The internal consistency coefficient of this scale in this study was 0.913. The internal motivation was measured using a scale developed by Ryan and Connell (1989), with examples like “I did this job because it was interesting.” In this study, the internal consistency coefficient of this scale was 0.857.

## Results

### Measurement tool description statistics

As can be seen from Table 1, the mean, standard deviation, skewness, and kurtosis of each variable and its question term, where the absolute value of skewness is less than two and the absolute value of kurtosis is less than seven, indicate that each variable basically obeys normal distribution.

**TABLE 1:** Description statistics of measurement tools.

Item and variable	*N*	Minimum	Maximum	Mean	Standard	Skewness	Kurtosis
Proactive personality mean	408	1.8	6.7	4.965	1.035	−1.004	1.204
FSSB mean	408	2	7	5.154	1.157	−0.747	0.360
Innovative behavior mean	408	1.375	6.688	4.631	1.298	−0.532	−0.858
Thriving at work mean	408	1.727	7	4.952	1.107	−0.554	0.009
Work-family enrichment mean	408	2	7	5.127	1.174	−0.971	0.566

### Validation factor analysis of discriminant validity among variables

To test whether the measurement variables in this study have convergent and discriminant validity, this study uses Mplus8.3 software to conduct confirmatory factor analysis on family supportive leadership behavior, work-family enrichment, teachers’ innovative behavior, teachers’ thriving at work and proactive personality. The analysis results showed that the five-factor model met the criteria (χ2/D F = 1.850, CFI = 0.919, TLI = 0.915, RMSEA = 0.046, SRMR = 0.041), and the fitting coefficients of the five-factor model were significantly better than those of the other models (see Table 2). The results in Table 3 show that the factor loadings of proactive personality, family-supportive supervisor behavior, innovative behavior, thriving at work, and work-family enrichment are above 0.5 and CR values are above 0.7, and all variables are above 0.5 except for proactive personality AVE value which is slightly below 0.5, so this questionnaire has good convergent validity. In Table 4 of the correlation coefficients, the square root of AVE for family supportive leadership behavior, proactive personality, work-family enrichment, innovative behavior and thriving at work are 0.767, 0.699, 0.764, 0.709, and 0.712, respectively, which are all greater than their corresponding correlation coefficients, thus indicating that the questionnaire has good discriminant validity.

**TABLE 2:** Confirmatory factor analysis.

Model	χ^2^	*df*	χ^2^/df	CFI	TLI	RMSEA	SRMR
Judgment criteria			<3	>0.9	>0.9	<0.08	0.08
(A/ B/C/D/E) Five-factor model (A/ B/C/D/E)	1729.687	935	1.850	0.919	0.915	0.046	0.041
(A+B/C/D/E) Four-factor model (A+B/C/D/E)	2224.772	939	2.369	0.870	0.863	0.058	0.049
(A+B+C/D/E) Three-factor model (A+B+C/D/E)	3072.332	942	3.261	0.784	0.773	0.074	0.088
(A+B+C+D/E) Two-factor model (A+B+C+D/E)	4826.817	944	5.113	0.606	0.587	0.100	0.121
(A+B+C+D+E) One-factor model (A+B+C+D+E)	6467.813	945	6.844	0.440	0.413	0.120	0.146

a: A, family supportive supervisor behavior; B, work-family enrichment; C, teachers’ innovation behavior, D, teachers’ thriving at work; E, proactive personality; b: “+” indicates that two factors are combined into one factor.

**TABLE 3:** Convergent validity.

Variables	Title	Factor load	S.E.	t-value	*P*-value	CR	AVE
Family-supportive supervisor behavior	X1	0.712	0.030	23.855	0.000	0.85	0.588
	X2	0.822	0.023	35.115	0.000		
	X3	0.857	0.022	39.684	0.000		
	X4	0.660	0.033	20.102	0.000		
Proactive personality	W1	0.823	0.019	43.035	0.000	0.905	0.489
	W2	0.787	0.022	35.998	0.000		
	W3	0.621	0.033	18.850	0.000		
	W4	0.626	0.033	19.153	0.000		
	W5	0.566	0.036	15.608	0.000		
	W6	0.709	0.027	25.886	0.000		
	W7	0.719	0.027	27.017	0.000		
	W8	0.662	0.030	21.792	0.000		
	W9	0.697	0.028	24.677	0.000		
	W10	0.745	0.025	29.924	0.000		
Innovative behavior	CXXW1	0.670	0.029	23.177	0.000	0.943	0.507
	CXXW2	0.700	0.027	26.014	0.000		
	CXXW3	0.748	0.023	31.915	0.000		
	CXXW4	0.757	0.023	33.233	0.000		
	CXXW5	0.720	0.026	28.189	0.000		
	CXXW6	0.746	0.024	31.581	0.000		
	CXXW7	0.753	0.023	32.494	0.000		
	CXXW8	0.705	0.027	26.594	0.000		
	CXXW9	0.717	0.026	27.909	0.000		
	CXXW10	0.708	0.026	26.931	0.000		
	CXXW11	0.667	0.029	22.911	0.000		
	CXXW12	0.691	0.027	25.155	0.000		
	CXXW13	0.699	0.027	25.986	0.000		
	CXXW14	0.696	0.027	25.649	0.000		
	CXXW15	0.708	0.026	26.904	0.000		
	CXXW16	0.699	0.027	25.945	0.000		
Thriving at work	GZFR1	0.635	0.032	19.998	0.000	0.917	0.502
	GZFR2	0.645	0.031	20.667	0.000		
	GZFR3	0.679	0.029	23.382	0.000		
	GZFR4	0.752	0.024	31.325	0.000		
	GZFR5	0.760	0.023	32.461	0.000		
	GZFR6	0.694	0.028	24.845	0.000		
	GZFR7	0.646	0.031	20.771	0.000		
	GZFR8	0.701	0.028	25.481	0.000		
	GZFR9	0.747	0.024	30.726	0.000		
	GZFR10	0.745	0.024	30.469	0.000		
	GZFR11	0.772	0.022	34.309	0.000		
Work-family enrichment	M1	0.712	0.030	23.614	0.000	0.847	0.584
	M2	0.845	0.023	37.377	0.000		
	M3	0.835	0.023	35.744	0.000		
	M4	0.645	0.035	18.495	0.000		

**TABLE 4:** Descriptive statistics and correlation analysis.

	M	SD	1	2	3	4	5	6	7	8	9	10	11
1. Gender	0.475	0.500	−										
2. Education	2.556	0.901	-0.027	−									
3. Age	2.350	1.055	0.000	0.068	−								
4. Years of work	2.610	1.297	0.048	-0.005	0.646**	−							
5. Internal motivation	5.134	1.170	0.015	0.032	-0.009	0.029	−						
6. Job requirements	4.456	0.717	-0.046	0.125*	-0.009	0.005	0.282**	−					
7. Family supportive leadership behavior	5.154	1.157	-0.023	0.003	-0.043	-0.043	0.349**	0.156**	0.767				
8. Proactive personality	4.965	1.035	0.097*	0.028	-0.102*	-0.02	0.242**	0.277**	0.122*	0.699			
9. Work-family enrichment	5.127	1.174	-0.015	0.064	0.005	0.023	0.328**	0.266**	0.409**	0.260**	0.764		
10. Thriving at work	4.952	1.107	0.012	0.05	-0.033	-0.039	0.399**	0.359**	0.363**	0.325**	0.469**	0.709	
11. Innovative behavior	4.631	1.298	-0.022	-0.004	0.03	0.041	0.347**	0.221**	0.281**	0.238**	0.304**	0.308**	0.712

*p < 0.05, **p < 0.01.

The diagonal is the square root of AVE.

### Descriptive statistics and correlation analysis

The means, standard deviations and correlation coefficients of each variable can be observed in Table 4. Among them, family-supportive supervisor behavior was positively and significantly correlated with work-family enrichment (*r* = 0.409, *p* < 0.01), with thriving at work (*r* = 0.363, *p* < 0.01), and with innovative behavior (*r* = 0.281, *p* < 0.01); work-family enrichment was positively and significantly correlated with thriving at work significantly (*r* = 0.469, *p* < 0.01) and work-family enrichment was positively correlated with innovative behavior significantly (*r* = 0.304, *p* < 0.01). The hypothesis was therefore tentatively supported.

### Hypothesis testing

**FIGURE 2:**
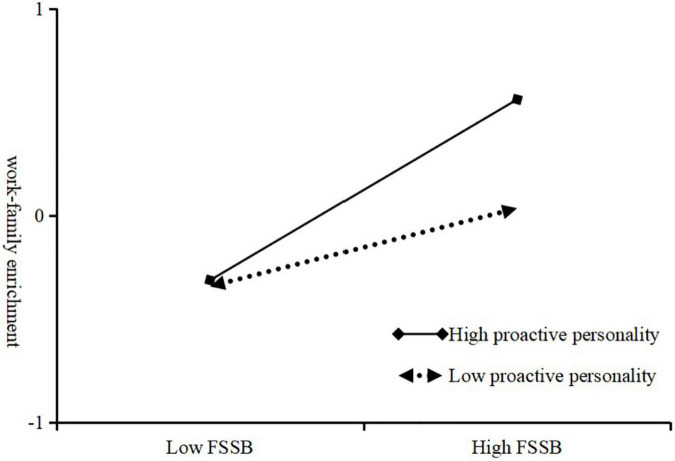
Analysis of the moderating effect of proactive personality on family supportive leadership and work-family enrichment.

In this study, SPSS 26.0 software was used for hierarchical regression analysis to test the hypotheses. In the cointegration test, the highest VIF value is 1.758, which is less than 10, thus indicating that there is no cointegration problem between the independent variables. It can be seen from Model 2 in Table 5 that family-supportive supervisor behavior has a significant positive effect on work-family enrichment (β = 0.327, *p* < 0.001). It can be seen from Model 4 in Table 5 that the interaction between family-supportive supervisor behavior and proactive personality has a significant positive effect on work-family enrichment (β = 0.124, *p* < 0.01), which indicates that proactive personality has a positive moderating effect between family-supportive supervisor behavior and work-family enrichment, and the hypothesis H4 is supported. It can be seen from Figure 2 that when the proactive personality is low, the positive effect of family supportive supervisor behavior on work-family enrichment is weak (β = 0.189, *t* = 2.856, *p* < 0.01), and when the proactive personality is high, the positive effect of family supportive supervisor behavior on work-family enrichment is weak (β = 0.189, *t* = 2.856, *p* < 0.01). Family-supportive supervisor behavior had a strong positive effect on work-family enrichment (β = 0.436, *t* = 7.098, *p* < 0.001).

**TABLE 5:** Test results of hierarchical regression analysis (with work-family enrichment as the dependent variable).

Variable	Variable work-family enrichment			
	**Model 1**	**Model 2**	**Model 3**	**Model 4**
**Control variables**				
Gender	-0.010	-0.003	v0.019	-0.005
Education	0.032	0.038	0.037	0.040
Age	-0.005	-0.002	0.019	0.018
Years of working experience	0.018	0.033	0.024	0.035
Internal motivation	0.275***	0.166***	0.141**	0.151**
Job requirements	0.184***	0.164***	0.128**	0.137**
**Independent variables**				
Family-supportive supervisor behavior		0.327***	0.322***	0.313***
**Moderating variables**				
Proactive personality			0.155***	0.139**
Independent variables*moderating variables				
Family-supportive supervisor behavior*proactive personality				0.124**
*R* ^2^	0.142	0.235	0.255	0.270
ΔR^2^	0.142	0.093***	0.021**	0.014**
F	11.029***	17.520***	17.116***	16.337***

With *p < 0.05, **p < 0.01, and ***p < 0.001, to obtain the standardized moderating effect, the data were standardized.

It can be seen from Table 6 that in Model 2, the family-supportive supervisor behavior has a significant positive effect on the teacher’s innovative behavior (β = 0.176, *p* < 0.001), and the hypothesis H1 is supported. In Model 3, family-supportive supervisor behavior has a significant positive impact on teachers’ innovative behavior (β = 0.126, *p* < 0.05), and work-family enrichment has a significant positive impact on innovative work behavior (β = 0.153, *p* < 0.01). Therefore, there is a partial mediating effect of work-family enrichment on the relationship between family-supportive supervisor behavior and teacher innovation behavior, and H3A is supported. In Model 5, family-supportive supervisor behavior has a significant positive effect on thriving at work (β = 0.237, *p* < 0.001), and the hypothesis H2 is supported. In Model 6, family-supportive supervisor behavior has a significant positive effect on thriving at work (β = 0.141, *p* < 0.01), and work-family enrichment has a significant positive effect on thriving at work (β = 0.294, *p* < 0.001), which indicates that work-family enrichment has a partial mediating effect on family-supportive supervisor behavior and thriving at work. Assume that H3B is supported.

**TABLE 6:** Test results of hierarchical regression analysis (with work-family enrichment as the dependent variable).

Variable	Innovative behavior			Thriving at work		
	**Model 1**	**Model 2**	**Model 3**	**Model 4**	**Model 5**	**Model 6**
**Control variables**						
Gender	-0.022	-0.018	-0.018	0.023	0.028	0.029
Education	-0.034	-0.031	-0.037	0.006	0.010	-0.001
Age	0.026	0.027	0.027	0.009	0.011	0.012
Years of working experience	0.016	0.024	0.019	-0.057	-0.046	-0.056
Internal motivation	0.310***	0.252***	0.226***	0.325***	0.246***	0.197***
Job requirements	0.137**	0.126**	0.101*	0.268***	0.253***	0.205***
**Independent variables**						
Family-supportive supervisor behavior		0.176***	0.126*		0.237***	0.141**
**Mediating variables**						
Work-family enrichment			0.153**			0.294***
R^2^	0.140	0.167	0.185	0.228	0.277	0.343
ΔR^2^	0.140	0.027***	0.018**	0.228	0.049***	0.066***
F	10.884***	11.451***	11.315***	19.774***	21.929***	26.088***

*p < 0.05, **p < 0.01, ***p < 0.001.

The mediating effect and the mediating effect with moderation were further tested by model 4 and model 7 of PROCESS 4.1, where Bootstrap was 5,000 times. As seen in Table 7, the mediating effect of work-family enrichment in family-supportive supervisor behavior and innovative work behavior is 0.050 with a confidence interval that does not contain 0 (0.013, 0.093), and hypothesis H3A is therefore further supported. The mediating effect of work-family enrichment in family-supportive supervisor behavior and thriving at work was 0.096, with a confidence interval not containing 0 (0.059, 0.138), and hypothesis H3B was further supported. At high and low values of proactive behavior, the mediating effects of work-family enrichment in family-supportive supervisor behavior and teacher innovation behavior were 0.029 and 0.067, with 95% confidence intervals of (0.003, 0.067) and (0.018, 0.123), respectively, both with significant mediating effects and a difference in mediating effects of 0.038, 95% confidence intervals did not contain 0 (0.007, 0.085). Therefore, it is suggested that proactive behavior moderates the mediating effect of work-family enrichment in family-supportive supervisor behavior and teacher innovation behavior, and hypothesis H5A is supported. The mediating effects of work-family enrichment in family-supportive supervisor behavior and thriving at work were 0.056 and 0.128, with 95% confidence intervals of (0.009, 0.104) and (0.082, 0.181), at high and low values of proactive behavior. Both mediating effects were significant, with a difference in mediating effects of 0.073 and 95% Confidence interval does not contain 0 (0.023, 0.135). It therefore indicates that proactive behavior moderates the mediating effect of work-family enrichment in family-supportive supervisor behavior and thriving at work, and hypothesis H5B is supported.

**TABLE 7:** Bootstrap results for mediating effects and mediated effects with moderation.

	Effect value	Standard error	Lower limit of confidence interval	Upper confidence interval
**Family-supportive supervisor behavior → work-family enrichment → innovative behavior**				
	0.050	0.021	0.013	0.093
**Family-supportive supervisor behavior → work-family enrichment → thriving at work**				
	0.096	0.021	0.059	0.138
**Family-supportive supervisor behavior → work-family enrichment → innovative behavior**				
Low proactive personality (−1SD)	0.029	0.017	0.003	0.067
High proactive personality (+1SD)	0.067	0.026	0.018	0.123
Difference	0.038	0.020	0.007	0.085
**Family-supportive supervisor behavior → work-family enrichment → Thriving at work**				
Low proactive personality (−1SD)	0.056	0.024	0.009	0.104
High proactive personality (+1SD)	0.128	0.025	0.082	0.181
Difference	0.073	0.028	0.023	0.135

## Discussion

### Research conclusions

Based on the work-home resource model and resource conservation theory, this paper explored the effects of family-supportive supervisor behaviors on teachers’ innovative behaviors and teachers’ thriving at work as well as the mechanisms of work-family enrichment and proactive personality in which the following conclusions were obtained. Family-supportive supervisor behavior have a significant positive predictive effect on teachers’ innovative behaviors and teachers’ thriving at work, and this effect is partially mediated through work-family enrichment. At the same time, proactive personality not only moderates the relationship between family-supportive supervisor behavior and work-family enrichment, but also further regulates the indirect relationship between family-supportive supervisor behavior affecting teachers’ innovative behaviors and teachers’ thriving at work through work-family enrichment. Specifically, the higher the level of proactive personality, the stronger the indirect effect of family-supportive supervisor behavior through work-family enrichment on teachers’ innovative behavior and teachers’ thriving at work.

### Theoretical implications

The theoretical contributions of this study mainly include the following four aspects. First, it responds to investigate the mechanism of family-supportive supervisor behavior on teachers’ innovative behavior and thriving at work from the perspective of work-family enrichment to fill the gaps in existing research on innovative behavior and thriving at work. School support has been found to have a significant impact on teachers’ work outcomes. However, these studies have mainly focused on examining the effects of formal support provided by schools to teachers in the work domain on innovative behaviors and thriving at work, neglecting to examine the effects of informal support provided by schools and managers from both the work and family ends on innovative behaviors and thriving at work and the mechanisms underlying them. Therefore, this study combined the work-home resource model and the resource conservation theory to study the influence path and mechanism of informal support of family-supportive supervisor behavior provided by managers from the perspective of work-family balance on teachers’ innovative work behavior and thriving at work from the positive interface of work-family enrichment. Secondly, this study fills some gaps in the current research in the work-family enrichment domain. Currently, although researchers have shown increasing interest in the work-family enrichment domain, they have mostly focused on the negative spillover effects (work-family conflict) between the work-family domain (Xin et al., 2018). Moreover, research on the antecedent and outcome variables of work-family enrichment is still poorly developed. Most studies have focused on whether individual psychological resources are conducive to work-family enrichment, but fewer studies have focused on the important role that supervisor support in the work domain can play in the achievement of positive work-family relationships (Carmeli and Russo, 2016). The present study incorporated work-family enrichment into the modeling framework and explored the mechanisms by which family-supportive supervisor behavior contribute to teachers’ innovative behaviors and thriving at work. This result enriches the research in the field of work-family enrichment and further complements the theory of work-home resources, thereby providing a reference and reference for subsequent related research. Finally, this study examined the moderating role of proactive personality in the process of family-supportive supervisor behavior influencing work-family enrichment and further explored the mediating effect of the first stage of being moderated. This contributes to the understanding of the specific mechanisms through which family-supportive supervisor behavior work, and enriches and deepens previous research based on the work-home resource model. Family-supportive supervisor behavior is a generator of resources; work-family enrichment is a transformer of resources; and proactive personality is a catalyst for resource generation and transformation. They work together to regulate the generation of family-supportive supervisor behavior (resources) and the transformation of work-family enrichment into resources. This suggests that the positive effects of proactive personality are not limited to mitigating work-family conflict, but can also facilitate supervisor support at work to promote teacher outcomes through work-family enrichment. At the same time, it also suggests to some extent that there are conditions for the application of the work-home resource model to be applicable. Specifically, the effects of family-supportive supervisor behavior on teachers’ innovative behaviors and thriving at work can vary at different levels of active personality. Therefore, future research should take note of the scope of application of the work-home resource model when applying it.

### Practical implications

The results of this study also have a multitude of implications for administrators and teachers. To begin with, based on the importance of family-supportive supervisor behaviors in influencing teachers’ thriving at work, we propose to enhance the application and cultivation of family-supportive supervisor behaviors in school management. Family supportive supervisor behavior is a form of informal organizational support provided by supervisors, and therefore the initiative and quality of such support can be further enhanced through appropriate training for supervisors and efforts to create a family supportive corporate culture. In addition, in the process of selecting supervisors, we should also examine whether the supervisor’s values endorse family-supportive behavior, and identify and select supervisors who approve of family-supportive behaviors as more beneficial to the school’s human resource management practices.

Secondly, the relationship between work and family is an issue of global concern. The higher the work-family enrichment of teachers, the more conducive it is to enhance the degree of innovation and investment of teachers. Therefore, schools should also shape teachers’ correct values of work and family through corporate culture, reduce teachers’ work pressure and improve teachers’ quality of family life. At the same time, exchanges such as training, lectures and salons are provided in schools to enhance teachers’ skills in dealing with work and family relations, guide teachers to find a balance between work and family step by step, and realize the positive effect of work on family by flexibly allocating their various material and psychological resources in the field of work and family. Therefore, teachers can make more efforts to obtain resources and stimulate teachers’ thriving at work and innovative behavior. In addition, flexible working system can also be implemented to meet the individual needs of teachers and fully mobilize the enthusiasm of teachers.

Finally, proactive personality plays an important role in determining the behavior of individuals. Teachers with high proactive personalities excel at communicating with people, building good relationships with supervisors, and inspiring more creativity and performance for the school with the support and encouragement of supervisors. Schools, especially innovative schools, can use proactive personality as a criterion for hiring, adding proactive personality tests to meet other hiring requirements and giving preference to those with high levels of proactive personality. For employees within the school, in addition to training teachers in creative and innovative skills, training can also target the initiative of supervisors and teachers to stimulate their potential proactive personalities and make them show more proactive behavior.

### Research shortcomings and prospects

This study has certain limitations and aspects that need to be studied in greater depth. Firstly, based on the work-home resource model and resource conservation theory, this study explored the relationship between family-supportive supervisor behavior and teachers’ innovative behaviors and thriving at work. However, the emotions and behaviors that individuals exhibit in their families are closely tied to the organizational context in which they are embedded; therefore, it is equally important to explore the impact of work context factors on teachers’ innovative work behaviors and thriving at work. To this end, we suggest that future research could further explore the effects of antecedent variables such as job requirements and feelings of school support on innovative work behaviors and thriving at work. Secondly, within the overall framework of the work-home resource model, this study explores the mechanisms underlying the influence between family-supportive supervisor behavior and teachers’ innovative behaviors and thriving at work, with strong theoretical contributions. However, the results of this study showed that work-family enrichment only partially mediated the effect. Therefore, future research can further enrich the existing findings by combining other theories (e.g., ecosystem theory, resource-acquisition-development model) to continue to explore other latent mediating mechanisms by which family-supportive supervisor behavior influences teachers’ innovative behavior and thriving at work. Finally, the use of a self-statement approach to data collection may lead to the problem of common method bias in this study. Although data were collected from three stages in this study, which could attenuate the problem of homoscedasticity to a certain extent, future studies could collect corresponding variables from different sources (e.g., having leaders fill out questionnaires on teachers’ innovative behavior and thriving at work) to improve the rigor of the research design.

## Data availability statement

The original contributions presented in this study are included in the article/Supplementary material, further inquiries can be directed to the corresponding author.

## Ethics statement

Ethical review and approval was not required for the study on human participants in accordance with the local legislation and institutional requirements. The patients/participants provided their written informed consent to participate in this study.

## Author contributions

QL responsible for article writing and data analysis. ML responsible for article revision and translation. Both authors listed have made a substantial, direct, and intellectual contribution to the work, and approved it for publication.

## Appendix

Dear Ms./Mr.

Hello! Thank you for taking the time to participate in this survey! This questionnaire is for academic research only, and the information you fill in is for the researcher’s use in scientific research. This questionnaire is anonymous, there is no right or wrong answers, the authenticity of your answers is very important to this study, your answers and information will be absolutely confidential! Please feel free to answer. Thank you for your cooperation and support!

I. Basic information (please cross “✓” on the corresponding option according to your real situation).

1. Your gender.

A Male B Female.

2. Your highest education.

A high school / junior college and below;

B College,

C Bachelor’s degree,

D Master and above,

3. Your age.

A Under 25 years old,

B 26–35 years old,

C 36–45 years old,

D 45 years old or above,

4. Your working years.

A 1 year and less,

B 1–3 years,

C 3–5 years,

D 5–10 years,

E 10 years and above,

Part 2

Please read the following entries carefully, each entry contains seven alternative answers, please answer according to your own true feelings and cross “✓” on the corresponding option, thank you for your cooperation!

1, Strongly disagree 2, Disagree 3, Basically disagree 4, General 5, Basically agree 6, Agree 7, Strongly agree.

1. I am constantly looking for new ways to improve my current life.

2. No matter where I am, I can always offer some constructive advice.

3. If I agree on something, I will do it no matter what the chance of success is.

4. I will stick to my ideas, even if someone opposes.

5. I will always look for the best way to deal with things.

6. When I firmly believe in an idea, no difficulty can stop me from implementing it.

7. I can find the opportunity earlier than others.

8. Few things are more exciting than seeing my ideas become reality.

9. If I see something I don’t like, I will try to change it.

10. I am good at finding new opportunities.

Part 3

Please read the following entries carefully, each entry contains seven alternative answers, please answer according to your own true feelings, thank you for your cooperation!

1, Strongly disagree 2, Disagree 3, Basically disagree 4, General 5, Basically agree 6, Agree 7, Strongly agree.

1. My boss makes me feel like I don’t have to worry about talking to him/her about work-life conflicts.

2. My supervisor works efficiently and is creative in resolving conflicts between work and non-work.

3. My supervisor demonstrates effective behavior regarding how to balance work and non-work issues.

4. My supervisor is able to organize work in the department/unit to the mutual benefit of the employee and the company.

Part 4

Please read the following entries carefully, each entry contains 7 alternative answers, please answer according to your own true feelings, thank you for your cooperation!

1, Strongly disagree 2, Disagree 3, Basically disagree 4, General 5, Basically agree 6, Agree 7, Strongly agree.

1. I encourage students to think about and discuss controversial issues.

2. I encourage students to use their creativity as much as possible in their learning.

3. I encourage students to explore new solutions to problems on their own.

4. I value students’ own thinking and summarizing of problems.

5. I advocate that students should have the courage to express views different from those of others.

6. I support students’ attempts and persistence on difficult tasks.

7. I often have ideas for reform and teaching innovation.

8. I use different teaching methods flexibly in order to impart knowledge and skills.

9. I want to try different forms of teaching organization.

10. I try to incorporate innovative ideas into my teaching activities.

11. I prepare a lot of extracurricular knowledge related to the teaching content from multiple perspectives.

12. I actively organize teaching activities that can enhance the interest of learning.

13. I take the initiative to learn the teaching experience of others.

14. I am open to others’ suggestions for improvement of my teaching.

15. I actively seek to learn from peers or experts and exchange opinions.

16. I pay attention to looking for feedback related to teaching from students’ studies.

Part 5

Please read the following entries carefully, each entry contains 7 alternative answers, please answer according to your own true feelings, thank you for your cooperation!

1, Strongly disagree 2, Disagree 3, Basically disagree 4, General 5, Basically agree 6, Agree 7, Strongly agree.

1. I am active and energetic in my work.

2. I have plenty of energy to complete my work.

3. My energy can last the whole day.

4. I have enough energy to complete the day’s work successfully.

5. When I go to work in the morning, I have enough energy to start a new day’s work.

6. When I work, I am in a state of mental invigoration.

7. The work I do can bring me positive energy.

8. I am able to maintain an energetic state at work.

9. I can learn many new things at work.

10. The new things I learn at work are helpful to my life.

11. The new things I learn at work make my life more exciting.

Part 6

Please read the following entries carefully, each entry contains 7 optional answers, please answer according to your own true feelings and cross “✓” on the corresponding option, thank you for your cooperation!

1, Strongly disagree 2, Disagree 3, Basically disagree 4, General 5, Basically agree 6, Agree 7, Strongly agree.

1. The work I do helps to deal with personal and practical problems at home.

2. The work I do allows me to become a more interesting person at home.

3. The day job goes well so that I can play the role of a partner better at home.

4. The skills I use at work are equally effective in dealing with family matters.

Part 7

There are many different reasons why everyone is in the job they currently have, so what motivated you to take it? Please read the following entries carefully, each entry contains 7 alternative answers, please answer according to your own true feelings, thank you for your cooperation!

1, Strongly disagree 2, Disagree 3, Basically disagree 4, Generally 5, Basically agree 6, Agree 7, Strongly agree.

1. I am in this job because it is interesting.

2. I am in this job because I like it very much.

3. I am engaged in this job because I enjoy this job brings me a sense of pleasure.

4. I am engaged in this job because the job itself is very attractive.

Part 8

Please read the following entries carefully, each entry contains five alternative answers, please answer according to the actual situation today and cross “✓” on the corresponding option, thank you for your cooperation!

1, very small 2, small 3, fair 4, large 5, very large.

1. How fast does your job require you to work.

2. How hard your job requires you to work.

3. How much your job requires a large workload.

4. How much your work time is not enough to complete the task.

5. How much your job requires you to work too much.

6. How much time you feel is not enough to complete your work.

7. How often your work requirements conflict with each other.

## References

[B1] Abid G. Arya B. Arshad A. Ahmed S. Farooqi S. (2021). Positive personality traits and self-leadership in sustainable organizations: Mediating influence of thriving and moderating role of proactive personality–science direct. *Sustain. Prod. Consumpt.* 25 299–311. 10.1016/j.spc.2020.09.005

[B2] Abid G. Sajjad I. Elahi N. S. Farooqi S. Nisar A. (2019). The influence of prosocial motivation and civility on work engagement: The mediating role of thriving at work. *Cogent Bus. Manage.* 5:1493712. 10.1080/23311975.2018.1493712

[B3] Ahmed I. Islam T. Afzal R. Iqbal I. Faheem M. A. (2023). Predicting the link between employees’ task performance and propensity to take charge: The role of family supportive supervision and LMX. *Int. J. Emerg. Mark.* 10.1108/IJOEM-12-2021-1859 [Epub ahead of print].

[B4] Aleksic I. Petkovic M. Jovanovic M. Milivojevic D. Vasiljevic B. Nikodinovic-Runic J. (2017). Anti-biofilm properties of bacterial di-rhamnolipids and their semi-synthetic amide derivatives. *Front. Microbiol.* 8:2454. 10.3389/fmicb.2017.02454 29276509 PMC5727045

[B5] Aryee S. Chu C. W. L. Kim T. Y. Ryu S. (2013). Family-supportive work environment and employee work behaviors: An investigation of mediating mechanisms. *J. Manage.* 39 792–813. 10.1177/0149206311435103

[B6] Atwater L. Carmeli A. (2009). Leader-member exchange, feelings of energy, and involvement in creative work. *Leadersh. Q.* 20 264–275. 10.1016/j.leaqua.2007.07.009

[B7] Aw S. S. Ilies R. Li X. Bakker A. B. Liu X. Y. (2021). Work-related helping and family functioning: A work–home resources perspective. *J. Occup. Organ. Psychol.* 94 55–79. 10.1111/joop.12331

[B8] Bakker A. B. Demerouti E. Dollard M. F. (2008). How job demands affect partners’ experience of exhaustion: Integrating work-family conflict and crossover theory. *J. Appl. Psychol.* 93 901–911. 10.1037/0021-9010.93.4.901 18642992

[B9] Bateman T. S. Crant J. M. (1993). The proactive component of organizational behavior: A measure and correlates. *J. Organ. Behav.* 14 103–118. 10.1002/job.4030140202

[B10] Bergeron D. M. Schroeder T. D. Martinez H. A. (2014). Proactive personality at work: Seeing more to do and doing more? *J. Bus. Psychol.* 29 71–86. 10.1007/s10869-013-9298-5

[B11] Boyar S. L. Mosley D. C. Jr. (2007). The relationship between core self-evaluations and work and family satisfaction: The mediating role of work-family conflict and facilitation. *J. Voc. Behav.* 71 265–281. 10.1016/j.jvb.2007.06.001

[B12] Carlson D. S. Thompson M. J. Crawford W. S. Kacmar K. M. (2019). Spillover and crossover of work resources: A test of the positive flow of resources through work–family enrichment. *J. Organ. Behav.* 40 709–722. 10.1002/job.2363

[B13] Carmeli A. Russo M. (2016). The power of micro-moves in cultivating regardful relationships: Implications for work–home enrichment and thriving. *Hum. Resour. Manage. Rev.* 26 112–124. 10.1016/j.hrmr.2015.09.007

[B14] Carmeli A. Spreitzer G. M. (2011). Trust, connectivity, and thriving: Implications for innovative behaviors at work. *J. Creat. Behav.* 43 169–191. 10.1002/j.2162-6057.2009.tb01313.x

[B15] Chan X. W. Kalliath P. Chan C. Kalliath T. (2020). How does family support facilitate job satisfaction? Investigating the chain mediating effects of work–family enrichment and job-related well-being. *Stress Health.* 36 97–104. 10.1002/smi.2918 31840406

[B16] Chang W. Busser J. A. (2020). Hospitality career retention: The role of contextual factors and thriving at work. *Int. J. Contemp. Hosp. Manage.* 32 193–211. 10.1108/IJCHM-10-2018-0831

[B17] Chen N. Zhang L. (2020). Mediating role of meaningful work and vocational identity on the relationship between perceived family supportive supervisor behaviour and career satisfaction. *J. Psychol. Afr.* 30 295–299. 10.1080/14330237.2020.1796024

[B18] Christensen-Salem A. Walumbwa F. O. Hsu C. I. C. Misati E. Babalola M. T. Kim K. (2021). Unmasking the creative self-efficacy–creative performance relationship: The roles of thriving at work, perceived work significance, and task interdependence. *Int. J. Hum. Resour. Manage.* 32 4820–4846. 10.1080/09585192.2019.1710721

[B19] Cinamon R. G. Rich Y. (2002). Gender differences in the importance of work and family roles: Implications for work–family conflict. *Sex Roles* 47 531–541. 10.1023/A:1022021804846

[B20] Crain T. L. Stevens S. C. (2018). Family-supportive supervisor behaviors: A review and recommendations for research and practice. *J. Organ. Behav.* 39 869–888. 10.1002/job.2320

[B21] Deng X. He S. Lyu P. Zhou X. Ye Y. Meng H. (2021). Spillover effects of workplace ostracism on employee family life: The role of need for affiliation and work-home segmentation preference. *Acta Psychol. Sin.* 53:1146. 10.3724/SP.J.1041.2021.01146

[B22] Du D. Bakker A. B. Derks D. (2020). Capitalization on positive family events and task performance: A perspective from the work–home resources model. *J. Occup. Health Psychol.* 25:357. 10.1037/ocp0000259 32718151

[B23] Erdogan D. T. Heras M. L. Rofcanin Y. Bosch M. J. Stollberger J. (2022). Family motivation of supervisors: Exploring the impact on subordinates’ work performance via family supportive supervisor behaviors and work–family balance satisfaction. *J. Appl. Soc. Psychol.* 52 1179–1195. 10.1111/jasp.12919

[B24] Gopalan N. Pattusamy M. Goodman S. (2021). Family incivility and work-engagement: Moderated mediation model of personal resources and family-work enrichment. *Curr. Psychol*. 10.1007/s12144-021-01420-4 [Epub ahead of print]. 33613014 PMC7887548

[B25] Greenhaus J. H. Powell G. N. (2006). When work and family are allies: A theory of work-family enrichment. *Acad. Manage. Rev.* 31 72–92. 10.5465/amr.2006.19379625

[B26] Hammer L. B. Ernst Kossek E. Bodner T. Crain T. (2013). Measurement development and validation of the Family Supportive Supervisor Behavior Short-Form (FSSB-SF). *J. Occup. Health Psychol.* 18 285–296. 10.1037/a0032612 23730803 PMC3863646

[B27] Hammer L. B. Kossek E. E. Yragui N. L. Bodner T. E. Hanson G. C. (2009). Development and validation of a multidimensional measure of family supportive supervisor behaviors (FSSB). *J. Manage.* 35 837–856. 10.1177/0149206308328510 21660254 PMC3109661

[B28] Hammer L. B. Wan W. H. Brockwood K. J. Bodner T. Mohr C. D. (2019). Supervisor support training effects on veteran health and work outcomes in the civilian workplace. *J. Appl. Psychol.* 104 52. 10.1037/apl0000354 30265016

[B29] Heskiau R. McCarthy J. M. (2021). A work–family enrichment intervention: Transferring resources across life domains. *J. Appl. Psychol.* 106:1573. 10.1037/apl0000833 33017156

[B30] Hildenbrand K. Sacramento C. Binnewies C. (2018). Transformational leadership and burnout: The role of thriving and followers’ openness to experience. *J. Occup. Health Psychol.* 23 31–43. 10.1037/ocp0000051 27631555

[B31] Horng J. S. Tsai C. Y. Yang T. C. Liu C. H. (2016). Exploring the relationship between proactive personality, work environment and employee creativity among tourism and hospitality employees. *Int. J. Hosp. Manage.* 54 25–34. 10.1016/j.ijhm.2016.01.004

[B32] Jiang H. Ma H. Y. Xie J. L. Zhang S. X. (2015). The effect of family-supportive supervisory behavior on employees’ work attitudes: A moderated mediation effect analysis. *Psychol. Sci.* 5 1194–1200.

[B33] Jiang Z. Jiang Y. Nielsen I. (2021). Thriving and career outcomes: The roles of achievement orientation and resilience. *Hum. Resour. Manage. J.* 31 143–164. 10.1111/1748-8583.12287

[B34] Kalliath P. Kalliath T. Chan X. W. Chan C. (2020). Enhancing job satisfaction through work–family enrichment and perceived supervisor support: The case of Australian social workers. *Pers. Rev.* 49 2055–2072. 10.1108/PR-06-2018-0219

[B35] Karasek R. (1979). Job decision latitude, job demands and mental strain: Implications for job redesign. *Adm. Sci. Q.* 24 285–308. 10.2307/2392498 30315367

[B36] Kim M. S. Ma E. Wang L. (2023). Work-family supportive benefits, programs, and policies and employee well-being: Implications for the hospitality industry. *Int. J. Hosp. Manage.* 108:103356. 10.1016/j.ijhm.2022.103356

[B37] Koekemoer E. Olckers C. Nel C. (2020). Work–family enrichment, job satisfaction, and work engagement: The mediating role of subjective career success. *Austral. J. Psychol.* 72 347–358. 10.1111/ajpy.12290

[B38] Kopperud K. H. Nerstad C. G. Dysvik A. (2020). Should i stay or should i go? The role of motivational climate and work–home spillover for turnover intentions. *Front. Psychol.* 11:1107. 10.3389/fpsyg.2020.01107 32581947 PMC7286056

[B39] Li C. Murad M. Shahzad F. Khan M. A. S. Dogbe C. S. K. (2020). Entrepreneurial passion to entrepreneurial behavior: Role of entrepreneurial alertness, entrepreneurial self-efficacy and proactive personality. *Front. Psychol.* 11:1611. 10.3389/fpsyg.2020.01611 32973593 PMC7468520

[B40] Liu B. Wang Q. Wu G. Zheng J. Li L. (2020). How family-supportive supervisor affect Chinese construction workers’ work-family conflict and turnover intention: Investigating the moderating role of work and family identity salience. *Construct. Manage. Econ.* 38 807–823. 10.1080/01446193.2020.1748892

[B41] Lu R. Wang Z. Lin X. Guo L. (2019). How do family role overload and work interferance with family affect the life satisfaction and sleep sufficiency of construction professionals? *Int. J. Environ. Res. Public Health* 16:3094. 10.3390/ijerph16173094 31454934 PMC6747144

[B42] Luo H. Li F. Agbanyo G. K. Tachega M. A. Chin T. (2022). Family-supportive supervisor behaviors and career sustainability of e-commerce female workers: A mixed-method approach. *Front. Psychol.* 13:992458. 10.3389/fpsyg.2022.992458 36237678 PMC9552821

[B43] Matthews R. A. Mills M. J. Trout R. C. English L. (2014). Family-supportive supervisor behaviors, work engagement, and subjective well-being: A contextually dependent mediated process. *J. Occup. Health Psychol.* 19 168–181. 10.1037/a0036012 24730426

[B44] McKersie S. J. Matthews R. A. Smith C. E. Barratt C. L. Hill R. T. (2019). A process model linking family-supportive supervision to employee creativity. *J. Occup. Organ. Psychol.* 92 707–735. 10.1111/joop.12276

[B45] Menges J. I. Tussing D. V. Wihler A. Grant A. M. (2017). When job performance is all relative: How family motivation energizes effort and compensates for intrinsic motivation. *Acad. Manage. J.* 60 695–719. 10.5465/amj.2014.0898

[B46] Olde-Dusseau H. N. (2012). Organizational Work-Family Resources as Predictors of Job Performance and Attitudes: The Process of Work-Family Conflict and Enrichment. *J. Occup. Health Psychol.* 17 28–40. 10.1037/a0026428 22149204

[B47] Olde-Dusseau H. N. Hammer L. B. Crain T. L. (2016). The influence of family-supportive supervisor training on employee job performance and attitudes: An organizational work-family intervention. *J. Occup. Health Psychol.* 21 296–308. 10.1037/a0039961 26652264

[B48] Porath C. Spreitzer G. Gibson C. Garnett F. G. (2012). Thriving at work: Toward its measurement, construct validation, and theoretical refinement. *J. Organ. Behav.* 33 250–275. 10.1002/job.756

[B49] Qaiser S. Abid G. Arya B. Farooqi S. (2020). Nourishing the bliss: Antecedents and mechanism of happiness at work. *Total Qual. Manage. Bus. Excell.* 31 1669–1683. 10.1080/14783363.2018.1493919

[B50] Rofcanin Y. Las Heras M. Escribano P. I. Stanko T. (2020). FSSBs and elderly care: Exploring the role of organizational context on employees’ overall health and work–family balance satisfaction. *J. Bus. Psychol.* 35 403–419. 10.1007/s10869-019-09629-8

[B51] Roshan T. Arulrajah A. A. (2021). The Influence of Family-Supportive Organization Perception and Work-Family Conflict on Absenteeism: The Mediating Role of Work Engagement. *IUP J. Organ. Behav.* 20 26–50.

[B52] Ryan R. M. Connell J. P. (1989). Perceived locus of causality and internalization: Examining reasons for acting in two domains. *J. Pers. Soc. Psychol.* 57 749–761. 10.1037/0022-3514.57.5.749 2810024

[B53] Seibert S. E. Crant J. M. Kraimer M. L. (1999). Proactive personality and career success. *J. Appl. Psychol.* 84 416–427. 10.1037/0021-9010.84.3.416 10380421

[B54] Shen C. Liu R. Yang J. Hu S. He P. (2022). How Family Supportive Supervisor Behaviors Enhance Employees’ Work-Family Enrichment? Thriving at Work as Mediator and Intrinsic Motivation as Moderator. *Psychol. Res. Behav. Manag.* 15 3133–3146. 10.2147/PRBM.S379000 36317091 PMC9617552

[B55] Shi Y. Xie J. Zhou Z. E. Tang H. Ma H. (2019). Family supportive supervisor behaviors and work engagement: A social information processing perspective. *Curr. Psychol.* 41 347–359. 10.1007/s12144-019-00574-6

[B56] Shi Y. Xie J. Zhou Z. E. Tang H. Ma H. Zhang H. (2020). Family-supportive supervisor behaviors and employees’ life satisfaction: The roles of work-self facilitation and generational differences. *Int. J. Stress Manage.* 27:262. 10.1037/str0000152

[B57] Spreitzer G. Sutcliffe K. Dutton J. Sonenshein S. Grant A. M. (2005). A socially embedded model of thriving at work. *Organ. Sci.* 16 537–549. 10.1287/orsc.1050.0153 19642375

[B58] Susanto P. Hoque M. E. Jannat T. Emely B. Zona M. A. Islam M. A. (2022). Work-life balance, job satisfaction, and job performance of SMEs employees: The moderating role of family-supportive supervisor behaviors. *Front. Psychol.* 13:906876. 10.3389/fpsyg.2022.906876 35800926 PMC9253617

[B59] Swanberg J. E. McKechnie S. P. Ojha M. U. James J. B. (2011). Schedule control, supervisor support and work engagement: A winning combination for workers in hourly jobs? *J. Voc. Behav.* 79 613–624. 10.1016/j.jvb.2011.04.012

[B60] Talukder A. M. H. (2019). Supervisor support and organizational commitment: The role of work–family conflict, job satisfaction, and work–life balance. *J. Employ. Counsel.* 56 98–116. 10.1002/joec.12125

[B61] Talukder A. M. H. Galang M. C. (2021). Supervisor support for employee performance in Australia: Mediating role of work-life balance, job, and life attitude. *J. Employ. Counsel.* 58 2–22. 10.1002/joec.12154

[B62] Ten Brummelhuis L. L. Bakker A. B. (2012). A resource perspective on the work-home interface: The work-home resources model. *Am. Psychol.* 67 545–556. 10.1037/a0027974 22506688

[B63] Timms C. Brough P. O’Driscoll M. Kalliath T. Siu O. L. Sit C. (2015). Flexible work arrangements, work engagement, turnover intentions and psychological health. *Asia Pac. J. Hum. Resour.* 53 83–103. 10.1111/1744-7941.12030

[B64] Van den Heuvel M. Demerouti E. Peeters M. C. (2015). The job crafting intervention: Effects on job resources, self-efficacy, and affective well-being. *J. Occup. Organ. Psychol.* 88 511–532. 10.1111/joop.12128

[B65] Wayne J. H. Grzywacz J. G. Carlson D. S. Kacmar K. M. (2007). Work–family facilitation: A theoretical explanation and model of primary antecedents and consequences. *Hum. Resour. Manage. Rev.* 17 63–76. 10.1016/j.hrmr.2007.01.002

[B66] Wayne J. H. Musisca N. Fleeson W. (2004). Considering the role of personality in the work-family experience: Relationships of the big five to work-family conflict and facilitation. *J. Voc. Behav.* 64 108–130. 10.1016/S0001-8791(03)00035-6

[B67] Xin J. Chen S. Kwan H. K. Chiu R. K. Yim F. H. K. (2018). Work-family spillover and crossover effects of sexual harassment: The moderating role of work-home segmentation preference. *J. Bus. Ethics* 147 619–629. 10.1007/s10551-015-2966-9

[B68] Yi-Feng Chen N. Crant J. M. Wang N. Kou Y. Qin Y. Yu J. (2021). When there is a will there is a way: The role of proactive personality in combating COVID-19. *J. Appl. Psychol.* 106 199–213. 10.1037/apl0000865 33600195

[B69] Yu A. Pichler S. Russo M. Hammer L. (2022). Family-supportive supervisor behaviors (FSSB) and work-family conflict: The role of stereotype content, supervisor gender, and gender role beliefs. *J. Occup. Organ. Psychol.* 95 275–304. 10.1111/joop.12379

[B70] Zhai Q. Wang S. Weadon H. (2020). Thriving at work as a mediator of the relationship between workplace support and life satisfaction. *J. Manage. Organ.* 26 1–17. 10.1017/jmo.2017.62

[B71] Zhang M. Zhang L. (2012). The relationship between teachers’ innovative work behaviors and innovative climate. *Hum. Ergon.* 18 1–6.

[B72] Zhang X. Bartol K. M. (2010). Linking empowering leadership and employee creativity: The influence of psychological empowerment, intrinsic motivation, and creative process engagement. *Acad. Manage. J.* 53 107–128. 10.5465/amj.2010.48037118

[B73] Zhao F. Q. Chen Y. Hu W. (2019). Research on impact of WFB-HRP on job performance in chinese context: Effect of family-work facilitation & psychological capital. *Nankai Bus. Rev.* 22 165–175.

[B74] Zhining W. Mengli L. Shu Z. (2020). The Multilevel Influence of Family-supportive Supervisory Behaviors on Employee Innovative Behavior–A Moderated Mediation Model. *Collect. Essays Finan. Econ.* 257:87.

[B75] Zhou X. Jin L. Wang Y. Liao W. Yang H. Li L. (2022). The influence of family supportive supervisor behavior on employee creativity: The mediating roles of psychological capital and positive emotion. *Front. Psychol.* 13:824840. 10.3389/fpsyg.2022.824840 35645879 PMC9133785

